# Capturing the clinical decision-making processes of expert and novice diabetic retinal graders using a ‘think-aloud’ approach

**DOI:** 10.1038/s41433-021-01554-6

**Published:** 2021-05-10

**Authors:** Katie Curran, Nathan Congdon, Tunde Peto, Catherine Dardis, Quan Nhu Nguyen, Tung Thanh Hoang, Finian Bannon, An Luu, Tung Quoc Mai, Van Thu Nguyen, Hue Thi Nguyen, Huong Tran, Hoang Huy Tran, Lynne Lohfeld

**Affiliations:** 1 Queens University Belfast, Centre for Public Health, Belfast, UK; 2 Orbis International, New York, NY USA; 3 Zhongshan Ophthalmic Centre, Sun Yat-sen University, Guangzhou, China; 4 Department of Ophthalmology, Belfast Health and Social Care Trust, Belfast, Northern Ireland; 5 Vitreo-Retina Department, Ho Chi Minh Eye Hospital, Ho Chi Minh City, Vietnam; 6 Ophthalmology Department, Hanoi Medical University, Hanoi, Vietnam; 7 Save Sight Institute, The University of Sydney School of Medicine, Camperdown, NSW Australia; 8 Orbis International, Hanoi, Vietnam; 9 Community Department, Ho Chi Minh Eye Hospital, Ho Chi Minh City, Vietnam; 10 Eye Hospital of Wenzhou Medical University, Wenzhou, Zhejiang China

**Keywords:** Retinal diseases, Medical imaging, Health occupations

## Abstract

**Background:**

Diabetic eye screening programmes have been developed worldwide based on evidence that early detection and treatment of diabetic retinopathy are crucial to preventing sight loss. However, little is known about the decision-making processes and training needs of diabetic retinal graders, particularly in low- and middle-income countries.

**Objectives:**

To provide data for improving evidence-based diabetic retinopathy training to help novice graders process fundus images more like experts.

**Subjects/methods:**

This is a mixed-methods qualitative study conducted in southern Vietnam and Northern Ireland. Novice diabetic retinal graders in Vietnam (*n* = 18) and expert graders in Northern Ireland (*n* = 5) were selected through a purposive sampling technique. Data were collected from 21st February to 3rd September 2019. The interviewer used neutral prompts during think-aloud sessions to encourage participants to verbalise their thought processes while grading fundus images from anonymised patients, followed by semi-structured interviews. Thematic framework analysis was used to identify themes, supported by illustrative quotes from interviews. Mann–Whitney *U* tests were used to compare graders’ performance.

**Results:**

Expert graders used a more systematic approach when grading images, considered all four images per patient and used available software tools such as red-free filters prior to making a decision on management. The most challenging features for novice graders were intra-retinal microvascular abnormalities and new vessels, which were more accurately identified by experts.

**Conclusion:**

Taking more time to grade fundus images and adopting a protocol-driven “checklist” approach may help novice graders to function more like experts.

## Introduction

Diabetes (DM) is one of the largest global health challenges of this century, affecting ~463 million adults aged 20–79 years old worldwide [1, 2]. According to the International Diabetes Federation, the adult prevalence of DM in Vietnam is 5.5%, nearly doubling over the past decade [2, 3]. Diabetic eye disease, which includes diabetic retinopathy (DR) and diabetic macular oedema, is the leading cause of visual impairment and blindness among working-age adults globally, affecting one-third of all people with DM [4–6].

Diabetic eye screening programmes (DESPs) have been developed worldwide in response to evidence showing that early detection and treatment of DR can prevent over 90% of sight loss [7–9]. In England and Wales, DR is no longer the leading cause of blindness among working-age adults since DESPs were fully implemented by 2008. Many countries have adopted the UK’s national DESP as a model [9–11]. The programme relies on a highly trained core of graders, often without medical backgrounds, who screen for the disease through protocol-driven review of fundus images of the retina, rather than through direct clinical examination [12].

Implementing DESPs in low- and middle-income countries (LMICs) can be challenging, and requires careful planning and evaluation [13]. In part, this is because many LMICs have minimal resources to screen and treat people with DR at both primary and secondary levels. Therefore, the risk for blindness among those with undiagnosed or poorly controlled DM can be high [14, 15]. Training cadres to provide efficient, user-friendly screening and treatment services is crucial in preventing and managing sight-threatening DR (STDR) [16]. Involving specially trained alternate cadres of graders allows task-shifting away from scarce and frequently overburdened ophthalmologists who can primarily spend time delivering treatment [17–20].

A key question, when training personnel to review fundus photos is how well novice graders carry out this important task. It would be useful to know how novice versus expert graders process information while grading images when developing training programmes for novice graders. Prior studies suggest that expert clinical decisions are typically based on pattern recognition, or the quick and efficient recall of previous cases, resulting in high diagnostic accuracy [21–23]. In contrast, novice graders tend to test data against learned hypotheses or diagnoses, which is a slower and more error-prone process. In addition, novice graders tend to take longer to complete tasks than experts, which suggests that they use additional processing cues to solve problems [24–26].

This study seeks to evaluate the clinical decision-making processes of expert and novice diabetic retinal graders in Vietnam and NI, and to use the findings to develop recommendations for more effective DR grader training programmes in Vietnam and other LMICs.

## Materials (subjects) and methods

### Study design

This mixed-methods study used multiple qualitative methods to collect data from a structured (simulation-based) interview with Likert-scale ratings during think-aloud (TA) sessions and from semi-structured interviews. The TA method entails systematically capturing participants’ statements about their thoughts while completing a task in order to evaluate their decision-making processes [27]. TA studies produce rich, in-depth data from small samples and are particularly useful if data are collected under realistic situations, such as grading actual fundus photographs in typical diabetic eye screening settings [28].

### Participants

Participants (23 diabetic retinal graders) were recruited from DESPs in five locations in southern Vietnam, supported by Orbis International, Queen’s University Belfast (QUB) and health care providers from the DESP in NI. The sample includes 14 novice graders (general nurses and newly qualified optometrists) and four experts (ophthalmologists) from Vietnam, plus five experts from NI (senior graders from the Belfast Ophthalmic Reading Centre). All invited graders participated in the study.

### Data collection

Two 45° field images using the Canon CR-2 digital fundus camera were taken per eye, one image centred on the optic disc and the other centred on the macula (image size 2736 × 1824). These were chosen to align with the UKs’ NHS DESP guidelines. Twenty-four macular and disc-centred images from six consenting patients in Vietnam (four images per patient, two per eye) were selected by a research optometrist from QUB (KC) from the database of the Orbis-supported DESP. The selected images represent a wide range of DR and maculopathy grades and warranted a range of responses ranging from annual re-screen to routine and urgent referrals. Interview guides were developed by researchers at QUB (KC, LL, TP, NC) based on their clinical and research experience. They were then translated from English to Vietnamese by bilingual Vietnamese-English speakers (AL, HTN, VTN) before pilot testing with a Vietnamese grader. Minimal changes were made to the interview guide based on the feedback.

Data were collected from 21st February to 3rd September 2019. After providing written informed consent, participants were given standardised written and oral instructions with non-ocular examples of what occurs during a TA interview, followed by a practice session that involved solving simple mathematics problems. All interviews were conducted in Vietnamese at the graders’ usual hospital settings by an English-speaking optometrist (KC) assisted by bilingual Vietnamese team members (AL, HTN, VTN). For each of the six sets of images, graders gave a DR and maculopathy grade; rated their difficulty in making that assessment on a 5-point Likert scale (1 = very difficult to 5 = very easy); made a recommendation as to appropriate follow-up care (annual screening, routine referral, or urgent referral); and rated their confidence in providing the recommendation on another 5-point Likert scale (1 = not confident at all to 5 = very confident). At the end of the TA sessions, semi-structured interviews were conducted by the same researchers to collect information on demographic variables, training experience, job roles and recommendations to improve future training. All interviews were recorded using a digital voice recorder (Sony ICD-BX140, Tokyo, Japan), the English portions transcribed verbatim by the interviewer (KC), and then reviewed by an expert qualitative researcher (LL) to ensure accuracy.

## Data analysis

### Qualitative data

After creating verbatim written transcripts of the translated audio files from interviews, a series of tables was created to compile quotes from each transcript related to a specific topic based on the interview guide [29, 30]. Two researchers (LL, KC) met on five occasions to discuss the data, assign codes and cluster them into themes, discussing all findings until consensus was reached. The standards for reporting qualitative research guidelines were closely followed [31].

### Quantitative data

Descriptive statistics were used to characterise the participants by age, sex and work experience. We assessed the data for normality using histograms, since the data were not normally distributed and the sample size was small (<30), data are presented as median and interquartile range (IQR). The non-parametric Mann–Whitney *U* test was used to compare differences between DR graders in NI versus Vietnam in terms of (a) time taken to grade six image sets, (b) self-reported difficulty and (c) self-reported confidence. Data were entered into an Excel spreadsheet and then analysed (KC, FB) using SPSS Statistics version 25.0 software (IBM, Armonk, NY).

To ensure the study was rigorous, a clear protocol was followed, and all steps were clearly documented. Prolonged visits to Vietnam and continuous communications with experts in the field were made. Triangulation of methods and of researchers were also used [32].

### Ethical issues

This research adhered to the tenets of the Declaration of Helsinki. Ethical approval was granted by the Hanoi Medical University Institutional Review Board in Vietnam and the Research Ethics Committee at QUB. Permission was also obtained from hospital directors at tertiary (Ho Chi Minh City General Hospital and Ho Chi Minh Eye Hospital), provincial (Tien Giang General Hospital) and district (Cai Ba General Hospital) level hospitals in Vietnam, where Vietnamese participants worked. Written informed consent was obtained from participants prior to recruitment.

### Data presentation

To promote readability of statements by study participants, quotes appear in italics; minor editing is shown with square brackets around added words and an ellipse to show where words have been removed. Participants were identified by source using a unique ID number, indicated by grader type, country and interview number e.g. nurse, Vietnam, GR1.

## Results

As expected, length of grading experience was greater among experts than novice graders, although the experts in NI grading images for five times longer than the experts in Vietnam (11.4 ± 10.9 years vs. 2.13 ± 1.93 years). In total, 21/23 (91.3%) of the graders in the study were female (Table 1).

**Table 1:** Demographic characteristics of participants (*n* = 23).

	Nurses (novices in VN)	Optometrists (novices in VN)	Ophthalmologists (experts in VN)	Senior graders (experts in NI)
Total, *n* (%)	11 (48)	3 (13)	4 (17)	5 (22)
Mean age, years (SD)	32.7 (5.53)	27 (7.81)	33.5 (32.0)	41.4 (6.69)
Female sex, *n* (%)	11 (100)	3 (100)	3 (75)	4 (80)
Mean grading experience, years (SD)	0.53 (0.47)	0.00 (0.00)	2.13 (1.93)	11.4 (10.9)

*VN* Vietnam, *NI* Northern Ireland, *SD* standard deviation.

Experts in NI spent longer time (median 24 min, 19 s ± IQR 23 min, 12 s to 54 min, 10 s) grading all six image sets compared to graders in Vietnam (17 min, 12 s ± 11 min, 38 s to 21 min, 1 s; *p* = 0.004). Table 2 shows no statistically significant differences between graders in NI and Vietnam in terms of self-reported difficulty for grading or confidence providing a recommended management plan.

**Table 2:** Median differences between graders from Vietnam versus graders from Northern Ireland in grading six fundus image sets.

	Graders Vietnam *n* = 18	Graders NI *n* = 5	*p* value
Time taken (min, s) to grade all image sets on average (median ± IQR)	17 min, 12 s (11 min, 38 s to 21 min, 1 s)	24 min 19 s (23 min, 12 s to 52 min, 10 s)	0.004
Self-reported difficulty grading (median ± IQR)	3.85 (3.24–4.34)	3.67 (3.67–4.08)	0.76
Confidence in providing outcome (median ± IQR)	3.96 (3.31–4.35)	4.33 (3.84–4.50)	0.35

Non-normal distribution and small sample size (*n * < 30); therefore, the non-parametric, Mann–Whitney *U* test was used to compare groups. Due to non-identical distributions for time taken, the *p* value must be interpreted with caution.

*IQR* interquartile range.

**Fig. 1:**
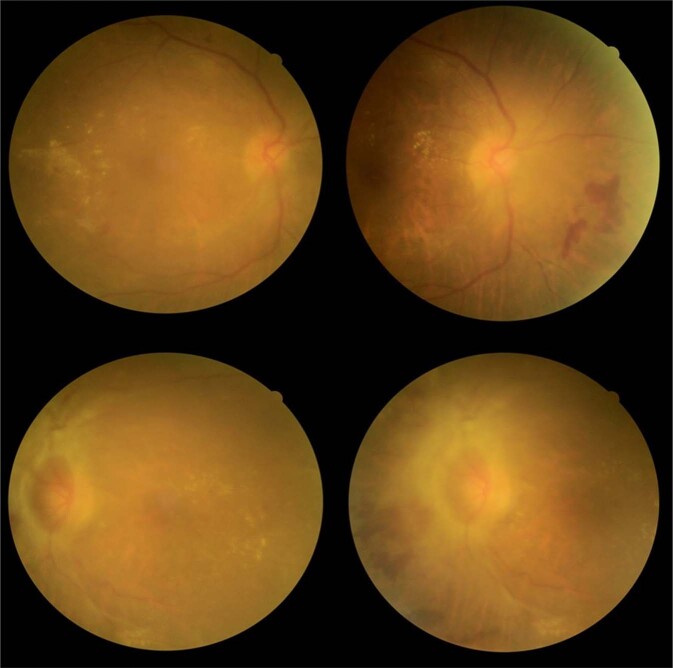
Image-set six showing signs of sight-threatening DR, partially obscured by cataract. The right eye as illustrated in the top two images shows signs of STDR. The images are somewhat unclear due to cataract. There is a pre-retinal haemorrhage, exudates and haemorrhages near the macula. There are new vessels superior to the disc. The bottom two images of the left eye also show obvious signs of proliferative DR. The disc is abnormal with fibrovascular changes, and a pre-retinal haemorrhage is present. There are vascular changes, exudates and haemorrhages.

Findings from two image sets are particularly noteworthy. Image set #6 shows obvious signs of STDR, despite some blurring due to cataract (Fig. 1). Yet only 36% (5/14) of the novice graders accurately assigned a grade and 64% (9/14, eight nurses and one optometrist) of them under-graded the image set. Despite the incorrect grading, 86% (12/14) of the graders, including all experts (9/9, 100%) in NI and Vietnam, correctly recommended an urgent referral for the patient.

**Fig. 2:**
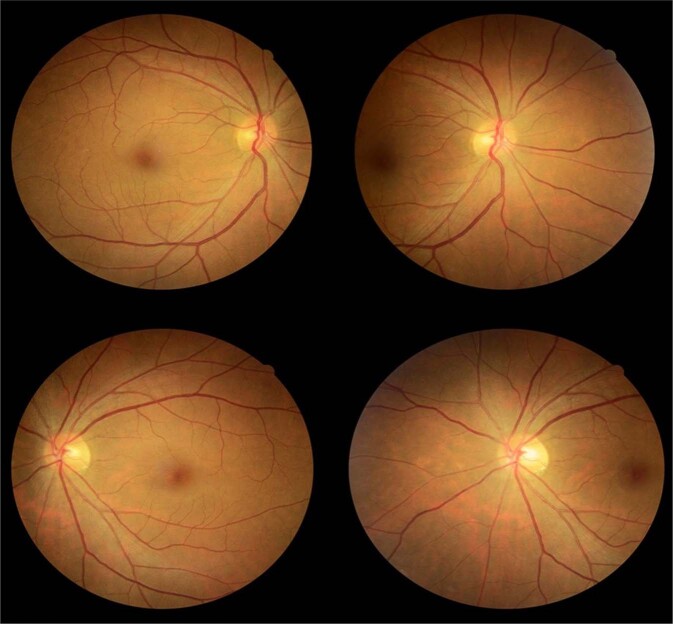
Image-set four showing no signs of DR. As seen from the four images above, the macular-centred and disc-centred images of the right eye are presented on top, and the macular-centred and disc-centred images of the left eye are presented below. No signs of DR are present in either eye.

Most novice graders in Vietnam (13/14 = 93%) accurately graded and recommended an annual re-screen for the patient in image set #4, which displayed no signs of DR (Fig. 2). However, one participant (1/14 = 7%) over-graded and over-managed the case, recommending a routine referral (within 3 months) rather than an annual re-screen. Although one expert grader in NI over-graded the image set, all experts (9/9 = 100%) correctly managed this case.

Different heuristic approaches to the task of grading fundus images between novice and expert graders were identified from the TA interview data. Experts tended to take a more systematic approach when grading images, examining the macular and disc images of both eyes before assigning a grade for the patient. In contrast, novice graders frequently graded the macular and disc images of each eye separately. Also, the novice graders tended to not comment on blood vessel appearance whereas expert graders, particularly those in NI, discussed the retinal vasculature in detail. Experts, but not novice graders, typically used all available software grading tools such as the red-free filter to reach their decision. As one expert grader from Vietnam explained, ‘The [red-free] filter [lets me] see the images more clearly’ (ophthalmologist, GR15). In contrast, an expert grader from NI reported using the filter for a specific purpose ‘[I will use] the red-free filter to look for signs of IRMA [intra-retinal microvascular abnormalities (IRMA)]’, (senior grader NI, GR18).

The TA interviews also provided data showing that all graders, regardless of level of experience, could accurately detect specific signs of DR such as microaneurysms, haemorrhages and exudates. However, the DR terminology used by experts was much more precise than what novice graders reported. As one novice grader explained, ‘There are white spots, but I can’t think of the name’ (nurse Vietnam, GR8). In contrast, an expert grader from Vietnam reported, ‘[In this image] we can see cotton wool spots’ (ophthalmologist Vietnam, GR14). Experts in Vietnam and NI were much better at identifying IRMA, fibrosis and new vessels compared to novice graders. Recommended management plans varied accordingly. An expert grader from Vietnam reported, ‘I can see some fibrovascular membrane, which is a sign of R3’ (ophthalmologist Vietnam, GR14) and an expert from NI stated, ‘There is fibrosis so [the DR] is quite severe’ (senior grader NI, GR19).

Data collected from the TA interviews also underscored the potential challenges, even for experts, of grading normal fundus image sets. As one senior grader from NI (senior grader NI, GR16) reported:

‘*It is…never…as easy to grade normal as abnormal* [images] *because you never really quite know if you missed something. And* [so] *usually I take twice as long to grade normal as abnormal* [images]*!*’

Graders in Vietnam reported being satisfied with their DR training course content. The main strength of the training for novice graders was their ability to develop new skills and better identify eye disease early, ‘It is really good that I can see damage to the eyes’, (nurse Vietnam, GR12); ‘To detect it [DR] early is better for patients so the patient can seek early treatment’, (nurse Vietnam, GR9). Novice graders clearly indicated that additional training would be beneficial, perhaps by being mentored by more experienced graders at the workplace (‘We can learn from others in discussions and case studies because further training takes up too much time’, (nurse Vietnam, GR11)). Non-medical graders in NI complete a mandatory, highly structured training programme and noted that there is value in learning to grade images oneself as for online courses (‘It makes your images a lot better when you have to grade them yourself’ (senior grader NI, GR17)).

## Discussion

To our knowledge, this is the first study to explore the clinical decision-making thought processes of DR graders in Vietnam. Expert graders in NI took more time to grade than other participants, and experts in both countries used a more systematic approach when grading images compared to novice graders. The systematic use of tools such as red-free filters was noted to be a crucial part of experts’ ability to detect more challenging findings such as IRMA, which was frequently missed by novice graders.

Contrary to our findings, previous studies have found that experts spend less time completing clinical tasks compared to novice graders [33–36]. Experts generally detect and identify problems more readily than novice graders; they notice specific features and make meaningful connections with information that may not be observed by novice graders [35]. In the current study, additional time required by experts in NI was partly due to their using a more systematic approach when grading fundus images, carefully examining all four photos in an image set and all features in each image, such as retinal vasculature. The shorter time spent by experts in Vietnam versus those in NI may reflect the fact that they regularly carry out multiple clinical responsibilities, reducing the usual time they could spend on grading images, even during this study.

According to the Early Treatment Diabetic Retinopathy Study, seven-field colour fundus photography is the gold standard for fundus imaging; however, this is time-consuming and potentially impractical for large-scale screening programmes. Imaging in single or multiple fields varies across countries, although most programmes use two field 45° field images, which is quicker and more convenient for patients. This protocol has been adopted by the UKs’ NHS DESP and was subsequently used in our study. The challenges of inconsistent DR programmes exist globally, but the concepts of basic DR grader training remain the same. Trainees should be reminded to examine fundus images systematically, review all of a patient’s photos and carefully assess retinal vasculature in each image before classifying DR disease. Zoom features and red-free filters are particularly useful to detect and confirm DR signs; therefore, consistent use of such grading tools is recommended. These insights are useful because training highly competent cadres of non-physician graders is particularly important in low-resource settings such as Vietnam, to allow ophthalmologists to focus more fully on delivering treatment.

Artificial intelligence (AI) and deep learning algorithms may significantly change future approaches to DR grading and are beginning to do so already in settings such as Scotland [37, 38]. However, continued attention to validating strategies that maximise the accuracy of human graders is still highly relevant in 2020, especially for low-resource areas where AI may not be available. Furthermore, AI systems have yet to be validated against a gold standard of proven human graders. Differences between the high-quality images used to train most existing systems and the types of images encountered in low-resource settings, with high rates of prevalent lens opacity, less-well-trained photographers and lower-cost cameras, mean that such validation must almost certainly occur at the local level. The continued importance of reliable human graders in low-resource settings is further underscored by the high cost of automated grading software, and the fact that few if any systems are able to function fully without input from human graders.

Strengths of the study include the enrolment of multiple cadres at different levels of training in two distinct settings, collecting data during TA interviews conducted in real-life workplace settings, following a carefully designed study protocol, involving an experienced qualitative researcher on the team and as a co-analyst, achieving thematic saturation from the interview data, and having selected relevant images of anonymous patients from an on-going DESP.

Limitations must also be acknowledged. The manner in which both NI and Vietnam participants graded fundus images in the current study may not completely reflect their typical work patterns: we did not use their usual grading software because it provides certain prompts. Instead, our goal was to document graders’ spontaneous approaches in order to understand their actual thought processes. Furthermore, application of these results outside of southern Vietnam and NI must be made with caution because the workplace context and culture may vary considerably from those in the present study.

In conclusion, this study provides insights into DR graders’ decision-making processes that highlight ways to improve their training. Training graders to achieve high sensitivity and high specificity is crucial to develop sustainable DESPs and prevent blindness in a population. Practical and interactive sessions were favourable among graders in Vietnam and evaluations of these approaches are more likely to lead to positive changes in grading processes and patient outcomes. Adopting a TA approach to train graders either in person or via online learning may be beneficial for the future [27, 39]. Since the outbreak of the recent pandemic, online learning has become increasingly more common. Providing feedback about how expert graders classify and manage DR could help novice graders to develop expert-like behaviours while understanding how to review and organise critical information needed to reach an accurate diagnosis and management plan. Furthermore, it would allow trainers to better understand trainees’ thinking processes while grading fundus images, particularly when dealing with complex cases. Workshops have been conducted internationally for clinicians to learn how to incorporate the TA method into training programmes, and feedback suggests that these methods may be effective [27]. Eye-tracking technology to assess differences in gaze behaviours between experts and novice graders could be included in upcoming DR educational interventions in Vietnam [40, 41]. A study in the UK reported that trainees demonstrated more uncertain gaze behaviours compared to consultants when interpreting images for DR [41]. The authors recommended that eye tracking would be beneficial for medical educational interventions in the future [41]. Regular meetings and discussions on DR cases in hospital settings in Vietnam, together with a range of novice and expert graders may be useful for quality improvement purposes. Globally, international test and training has been fundamental for tracking graders progress and enhancing their skills. Other techniques that may be valuable to increase grading quality include clinical audits, development of country-specific DR guidelines, strong leadership and performance management approaches, all of which may be relevant to Vietnam. Further research to explore these recommendations are required.

## Summary

### What was known before

- No previous TA studies have been conducted previously to assess diabetic retinal graders’ decision-making processes.
- Non-ophthalmologists have successfully been trained in DR screening and grading in high-income settings.

### What this study adds

- Expert graders in Northern Ireland take longer to grade images for DR severity compared to diabetic retinal graders in Vietnam, ensuring that no DR features are missed.
- Expert graders use a more systematic approach to grade fundus images for DR compared to novices.
- Findings from this study have been incorporated into existing diabetic eye screening training programmes in Vietnam.
